# Crystal structure of tris­(4,7-diphenyl-1,10-phenanthroline-κ^2^ *N*,*N*′)cobalt(III) tris­(hexa­fluoro­phosphate) monohydrate

**DOI:** 10.1107/S2056989022001359

**Published:** 2022-02-10

**Authors:** Asma Mani, Jamel Eddine Belgaied, Gilles Gasser, Olivier Blacque

**Affiliations:** a Carthage University, National Institute of Applied Sciences and Technology, EcoChimie Laboratory, Tunis, Tunisia; b Chimie ParisTech, PSL University, CNRS, Institute of Chemistry for Life and, Health Sciences, Laboratory for Inorganic Chemical Biology, F-75005 Paris, France; c Department of Chemistry, University of Zurich, Winterthurerstrasse 190, CH-8057, Zurich, Switzerland

**Keywords:** crystal structure, cobalt(III) complexes, bathophenanthroline, photodynamic therapy, photodynamic therapy

## Abstract

The title compound, [Co(C_72_H_48_N_6_)](PF_6_)_3_·H_2_O, crystallizes with one tripositive complex mol­ecule, three hexa­fluoro­phosphate anions and one solvent mol­ecule of water in the asymmetric unit. The N_6_ coordination set around the central Co^III^ atom defines a distorted octa­hedral environment. Four fluorine atoms of one hexa­fluoro­phosphate anion are disordered over two sets of positions with site-occupancy factors of 0.697 (5) and 0.303 (5). In the crystal, inter­molecular π–π stacking inter­actions, C—H⋯π, C—H⋯F and O—H⋯F and inter­actions are present.

## Abstract 2

The title compound acts as a potential photosensitizer in photodynamic therapy against tumoral cells. It co-crystallizes with one solvent mol­ecule of water. In the crystal, inter­molecular π–π stacking inter­actions, C—H⋯π, C—H⋯F, and O—H⋯F and inter­actions are present.

## Chemical context

Over the years, metal complexes with polypyridyl ligands have been investigated as photosensitizers in photodynamic therapy (PDT) against cancer. Ru^II^ remains undoubtedly the most studied metal for this purpose due to its tunable photophysical properties (Caspar *et al.*, 2006 ▸; Howerton *et al.*, 2012 ▸; Heinemann *et al.*, 2017 ▸; Monro *et al.*, 2019 ▸; McFarland *et al.*, 2020 ▸). 
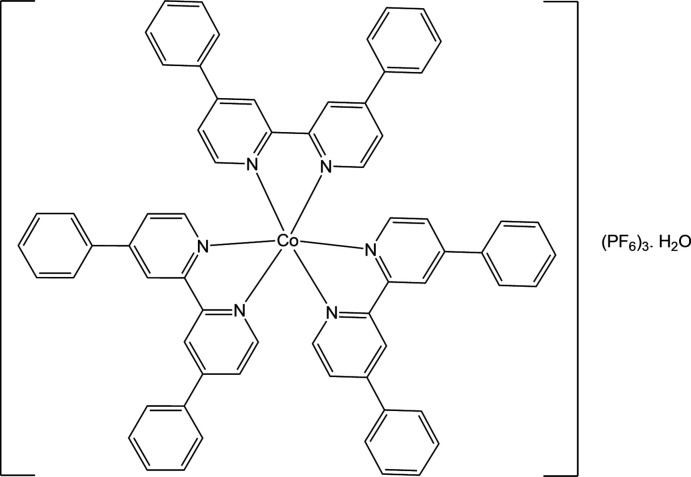


Inspired by the exciting results reported with Ru^II^, we were motivated to develop new metal-based complexes with similar structures. Among the transition metals, cobalt is commonly known for its potential to coordinate with chelate ligands like amino-acid compounds (Otter & Hartshorn, 2004 ▸) and polypyridyl derivative ligands. The resulting compounds were used in different fields of research. A series of Co^III^ complexes based on substituted 3-(pyridine-2-yl)-triazine ligands (Wang *et al.*, 2004 ▸), or bis­(1,10-phenanthroline), bis­(2,2′-bi­pyridine) and derivatized imidazole-phenanthroline ligands were developed (Nagababu *et al.*, 2008 ▸). These compounds were found to cleave calf thymus DNA (Zhang *et al.*, 2001 ▸).

Cobalt complexes are not only used for biological purposes. For example, a series of substituted polypyridine ligands, acting in a bidentate or tridentate manner, coordinating to Co^II^ were investigated as electron-transfer mediators in dye-sensitized solar cells (Sapp *et al.*, 2002 ▸). Tris(2,2′-bipyrid­yl)-based ligands were also used to design redox stable Co^II/III^ complexes for redox flow batteries (Yang *et al.*, 2018 ▸).

Encouraged by these results, our team aimed at developing new cobalt complexes. Here we report on the synthesis and crystal structure of [tris­(4,7-diphenyl-1,10-phenanthroline) cobalt(III)] tris (hexa­fluoro­phosphate) monohydrate, [Co^III^(C_72_H_48_N_6_)]^3+^(PF_6_^−^)_3_·H_2_O.

## Structural commentary

**Figure 1:**
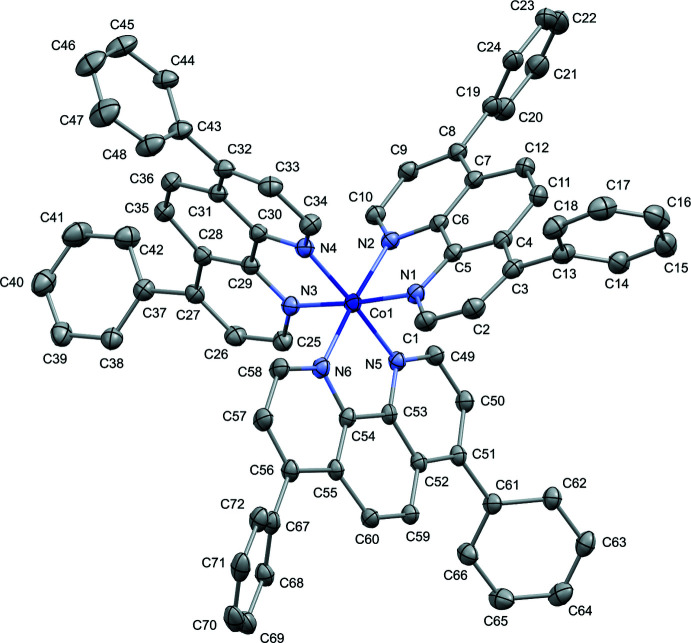
The mol­ecular structure of the tris­(4,7-diphenyl-1,10-phenanthroline)cobalt(III) cation of the title compound with displacement ellipsoids drawn at the 30% probability level. Hydrogen atoms are omitted for clarity.

The shape of the cobalt complex in the title compound is pseudo­octa­hedral (Fig. 1 ▸). The cobalt(III) atom is coordinated by six nitro­gen atoms from three dip ligands (dip = 4,7-diphenyl-1,10-phenanthroline). The Co—N bond lengths are in the range 1.934 (3)–1.954 (3) Å (Table 1 ▸) and correlate well with literature values observed for Co^III^ species. Indeed, the average Co—N bond length is 2.128 Å in Co^I^ cations (three hits in the Cambridge Structural Database (CSD; Groom *et al.*, 2016 ▸), 2.115 Å in Co^II^ cations (106 hits), and 1.952 Å in Co^III^ cations (28 hits) in reported Co(phen)_3_^*n*+^ (phen = phenanthroline) species. The bond angles between the axially bound ligand atoms are in the range 175.62 (13)–176.52 (13)° while the equatorial bond angles fall in the range 83.36 (12)–94.01 (13)°. The phenanthroline moieties (14 non-hydrogen atoms) of the dip ligands are almost planar according to the r.m.s. deviations calculated as 0.026 (N1^N2 moiety), 0.057 (N5^N6) and 0.106 (N3^N4) Å. As expected, the dihedral angles between the mean planes of the dip ligands are relatively close to 90° being 78.97 (5), 81.30 (4) and 86.09 (5)°. The phenyl rings substituting each phenanthroline ligand in *para* positions to the nitro­gen atoms exhibit an inter­mediate orientation (45–60°) relative to the mean plane of the phenanthroline ring. The dihedral angles between the mean planes are 65.91 (13) and 46.44 (13)° within the N1^N2 ligand, 50.37 (12) and 60.35 (14)° within the N3^N4 ligand, and 54.66 (14) and 42.35 (14)° within the N5^N6 ligand.

## Supra­molecular features

**Figure 2:**
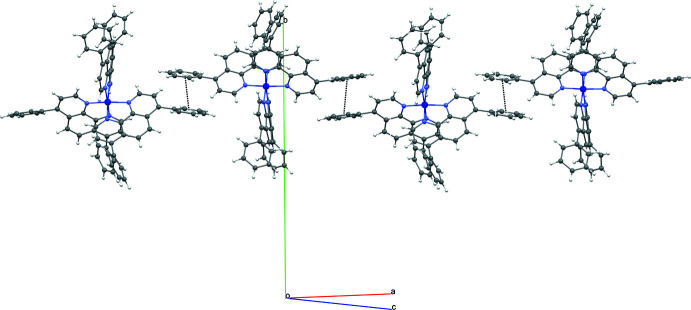
A view of the crystal packing showing π–π stacking inter­actions forming chains extending perpendicular to the *b* axis.

**Figure 3:**
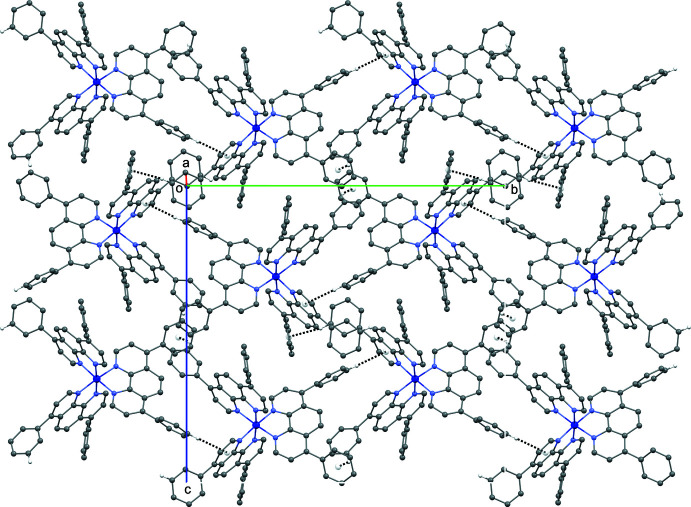
A view of the crystal packing along the *a* axis. The C—H⋯π hydrogen bonds are shown as dashed lines.

**Figure 4:**
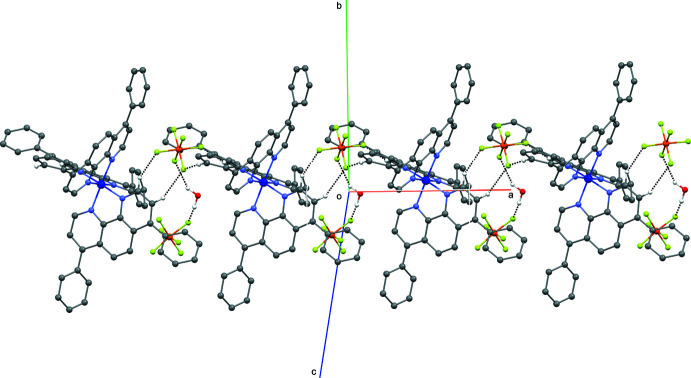
A view of the crystal packing showing C—H⋯F and O—H⋯F inter­molecular hydrogen bonds forming chains along the *a* axis. For clarity, only the major occupancy component of the disordered PF_6_^−^ anion is shown.

In the crystal, the complex cationic species inter­act with each other through π–π stacking inter­actions, forming chains extending perpendicular to the the *b* axis [*Cg*1⋯*Cg*2(1 + *x*, 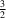 − *y*, 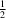 + *z*) centroid-to-centroid distance of 3.707 (3) Å with *Cg*1 being the centroid of atoms C19–C24 and *Cg*2 the centroid of atoms C67–C72; Fig. 2 ▸, Table 2 ▸] and C—H⋯π inter­actions, forming layers parallel to the *bc* plane (Fig. 3 ▸, Table 2 ▸). Weak C—H⋯F and classical O—H⋯F inter­molecular hydrogen bonds link the anionic hexa­fluoro­phosphate species (acceptors) to the tricationic mol­ecules and to the solvent water mol­ecules (donors). These inter­actions form chains along the *a* axis (Fig. 4 ▸). The most significant inter­actions for which C⋯F < 3.35 Å and C—H⋯F > 125°, and O⋯F < 3.00 Å and O–H⋯F > 125° are complied in Table 2 ▸.

### Database survey

A search of the CSD (version 5.43, last updated November 2021; Groom *et al.*, 2016 ▸) for similar *M*(dip)_3_^*n*+^ compounds gave three hits: two compounds with Ru^II^ as the central metal cation (*n* = 2; CSD refcodes LAKCIN: Alatrash & Macdonnell, 2020 ▸; DOWREM: Goldstein *et al.*, 1986 ▸) and one compound with Ni^II^ (*n* = 2; refcode EYAHUI: Hadadzadeh *et al.*, 2011 ▸).

## Synthesis and crystallization

[Tris(4,7-diphenyl-1,10-phenanthroline)cobalt(III)] tris(hexa­fluoro­phosphate) was obtained following the proc­edure previously described (McLaurin *et al.*, 2009 ▸). The experimental protocol used for the synthesis has two steps: Firstly, the synthesis of the [bis­(4,7-diphenyl-1,10-phenanthroline)cobalt(III) dichloride] chloride was carried out by the reaction of (4,7-diphenyl-1,10-phenanthroline) with cobalt(II) dichlor­ide in methanol at reflux. The obtained compound was oxidized with chlorine gas made *in situ* to convert Co^II^ to Co^III^. Finally, the substitution of the dichloride group for the bidentate ligand (4,7-diphenyl-1,10-phenanthroline) was performed in ethyl­ene glycol at reflux. After cooling to room temperature, ammonium hexa­fluoro­phosphate was added to obtain a dark-brown precipitate. The final complex was then isolated by filtration, washed with water and diethyl ether and dried under vacuum. Slow diffusion between methanol and diethyl ether of the aceto­nitrile solution of the obtained powder gave orange needles of the title compound suitable for X-ray diffraction.

## Refinement

Crystal data, data collection and structure refinement details are summarized in Table 3 ▸. The C—H hydrogen atoms were positioned geometrically with C—H = 0.95 Å and refined with *U*_iso_(H) = 1.2*U*_eq_(C). The O—H hydrogen atoms were located in a difference-Fourier map, but their positional and isotropic displacement parameters were restrained with the *SHELXL* DFIX command and with *U*_iso_(H) = 1.5*U*_eq_(O), respectively. Four fluorine atoms of one hexa­fluoro­phosphate anion (P3 as the central atom) are disordered over two sets of positions with refined site-occupancy factors of 0.697 (5) and 0.303 (5). The corresponding P—F bond lengths and F—P—F bond angles were restrained with the *DFIX* and *DANG* commands while the displacement parameters were restrained with the *SIMU* command.

## Supplementary Material

Crystal structure: contains datablock(s) I. DOI: 10.1107/S2056989022001359/wm5634sup1.cif

Structure factors: contains datablock(s) I. DOI: 10.1107/S2056989022001359/wm5634Isup2.hkl

CCDC reference: 2149884

Additional supporting information:  crystallographic information; 3D view; checkCIF report

## Figures and Tables

**Table 1:** Selected geometric parameters (Å, °)

Co1—N1	1.950 (3)	Co1—N4	1.942 (3)
Co1—N2	1.954 (3)	Co1—N5	1.941 (3)
Co1—N3	1.934 (3)	Co1—N6	1.940 (3)
			
*Cg*1⋯*Cg*2^i^	3.707 (3)		
			
N1—Co1—N2	83.72 (13)	N5—Co1—N1	88.67 (13)
N3—Co1—N1	175.62 (13)	N5—Co1—N2	93.26 (12)
N3—Co1—N2	92.66 (13)	N5—Co1—N4	176.52 (13)
N3—Co1—N4	84.01 (13)	N6—Co1—N1	93.44 (13)
N3—Co1—N5	94.01 (13)	N6—Co1—N2	175.65 (13)
N3—Co1—N6	90.31 (13)	N6—Co1—N4	93.77 (13)
N4—Co1—N1	93.48 (13)	N6—Co1—N5	83.36 (12)
N4—Co1—N2	89.70 (13)		

Symmetry code: (i) 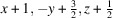 .

**Table 2:** Hydrogen-bond geometry (Å, °) *Cg*1, *Cg*2, *Cg*3 and *Cg*4 are the centroids of atoms C19–C24, C67–C72, C37–C42 and N5/C49–C53, respectively.

*D*—H⋯*A*	*D*—H	H⋯*A*	*D*⋯*A*	*D*—H⋯*A*
C9—H9⋯F7^ii^	0.95	2.46	3.300 (5)	148
C10—H10⋯F9^ii^	0.95	2.33	3.173 (5)	148
C25—H25⋯F1	0.95	2.45	3.204 (5)	136
C42—H42⋯F15*B* ^iii^	0.95	2.36	3.096 (7)	134
C48—H48⋯F7	0.95	2.39	3.328 (6)	172
C49—H49⋯F18*A* ^ii^	0.95	2.13	2.850 (9)	132
C58—H58⋯F12	0.95	2.26	2.963 (5)	130
O1—H1*A*⋯F17*A*	0.87 (1)	2.25 (7)	2.965 (17)	139 (9)
O1—H1*A*⋯F17*B*	0.87 (1)	2.19 (8)	2.817 (10)	128 (8)
O1—H1*B*⋯F11	0.87 (1)	2.28 (7)	2.977 (7)	137 (8)
C17—H17⋯*Cg*3^iv^	0.95	2.80	3.525 (6)	134
C46—H46⋯*Cg*4^iii^	0.95	2.72	3.670 (6)	177
C63—H63⋯*Cg*5^v^	0.95	2.59	3.466 (5)	154

Symmetry codes: (ii) 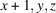 ; (iii) 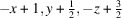 ; (iv) 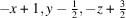 ; (v) 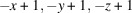 .

**Table 3:** Experimental details

Crystal data	
Chemical formula	[Co(C_24_H_16_N_2_)_3_](PF_6_)_3_·H_2_O
*M*_r_	1509.02
Crystal system, space group	Monoclinic, *P*2_1_/*c*
Temperature (K)	160
*a*, *b*, *c* (Å)	11.23448 (10), 25.0698 (2), 23.3956 (2)
β (°)	96.9903 (8)
*V* (Å^3^)	6540.29 (10)
*Z*	4
Radiation type	Cu *K*α
μ (mm^−1^)	3.66
Crystal size (mm)	0.18 × 0.12 × 0.02

Data collection	
Diffractometer	XtaLAB Synergy, Dualflex, Pilatus 200K
Absorption correction	Analytical [(*CrysAlis PRO*; Rigaku OD (2019 ▸) based on expressions derived by Clark & Reid, 1995 ▸]
*T*_min_, *T*_max_	0.595, 0.929
No. of measured, independent and observed [*I* > 2σ(*I*)] reflections	71503, 13335, 11500
*R*_int_	0.040
(sin θ/λ)_max_ (Å^−1^)	0.625

Refinement	
*R*[*F* ^2^ > 2σ(*F* ^2^)], *wR*(*F* ^2^), *S*	0.080, 0.243, 1.04
No. of reflections	13335
No. of parameters	953
No. of restraints	272
H-atom treatment	H atoms treated by a mixture of independent and constrained refinement
Δρ_max_, Δρ_min_ (e Å^−3^)	1.88, −1.05

Computer programs: *CrysAlis PRO* (Rigaku OD, 2019 ▸), *SHELXT* (Sheldrick, 2015*a* ▸), *SHELXL* (Sheldrick, 2015*b* ▸), *OLEX2* (Dolomanov *et al.*, 2009 ▸) and *publCIF* (Westrip, 2010 ▸).

## supplementary crystallographic information

### Crystal data

**Table:**

[Co(C_24_H_16_N_2_)_3_](PF_6_)_3_·H_2_O	*F*(000) = 3064
*M**_r_* = 1509.02	*D*_x_ = 1.533 Mg m^−^^3^
Monoclinic, *P*2_1_/*c*	Cu *K*α radiation, λ = 1.54184 Å
*a* = 11.23448 (10) Å	Cell parameters from 32214 reflections
*b* = 25.0698 (2) Å	θ = 2.6–78.7°
*c* = 23.3956 (2) Å	µ = 3.66 mm^−^^1^
β = 96.9903 (8)°	*T* = 160 K
*V* = 6540.29 (10) Å^3^	Plate, yellow
*Z* = 4	0.18 × 0.12 × 0.02 mm

### Data collection

**Table:**

XtaLAB Synergy, Dualflex, Pilatus 200K diffractometer	13335 independent reflections
Radiation source: micro-focus sealed X-ray tube, PhotonJet (Cu) X-ray Source	11500 reflections with *I* > 2σ(*I*)
Mirror monochromator	*R*_int_ = 0.040
Detector resolution: 5.8140 pixels mm^-1^	θ_max_ = 74.5°, θ_min_ = 2.6°
ω scans	*h* = −14→13
Absorption correction: analytical [(CrysAlisPro; Rigaku OD (2019) based on expressions derived by Clark & Reid, 1995]	*k* = −31→31
*T*_min_ = 0.595, *T*_max_ = 0.929	*l* = −29→26
71503 measured reflections	

### Refinement

**Table:**

Refinement on *F*^2^	Primary atom site location: dual
Least-squares matrix: full	Hydrogen site location: mixed
*R*[*F*^2^ > 2σ(*F*^2^)] = 0.080	H atoms treated by a mixture of independent and constrained refinement
*wR*(*F*^2^) = 0.243	*w* = 1/[σ^2^(*F*_o_^2^) + (0.1493*P*)^2^ + 10.0383*P*] where *P* = (*F*_o_^2^ + 2*F*_c_^2^)/3
*S* = 1.04	(Δ/σ)_max_ = 0.002
13335 reflections	Δρ_max_ = 1.88 e Å^−^^3^
953 parameters	Δρ_min_ = −1.05 e Å^−^^3^
272 restraints	

### Special details

**Table:**

Geometry. All esds (except the esd in the dihedral angle between two l.s. planes) are estimated using the full covariance matrix. The cell esds are taken into account individually in the estimation of esds in distances, angles and torsion angles; correlations between esds in cell parameters are only used when they are defined by crystal symmetry. An approximate (isotropic) treatment of cell esds is used for estimating esds involving l.s. planes.

### Fractional atomic coordinates and isotropic or equivalent isotropic displacement parameters (Å^2^)

**Table:**

	*x*	*y*	*z*	*U*_iso_*/*U*_eq_	Occ. (<1)
C1	0.4968 (4)	0.63319 (16)	0.71887 (18)	0.0419 (8)	
H1	0.436429	0.642234	0.688364	0.050*	
C2	0.4754 (4)	0.59200 (17)	0.75643 (18)	0.0447 (9)	
H2	0.400029	0.574379	0.751734	0.054*	
C3	0.5613 (4)	0.57648 (15)	0.79997 (17)	0.0401 (8)	
C4	0.6716 (3)	0.60482 (14)	0.80685 (16)	0.0366 (7)	
C5	0.6857 (3)	0.64682 (14)	0.76893 (15)	0.0337 (7)	
C6	0.7914 (3)	0.67862 (14)	0.77523 (15)	0.0330 (7)	
C7	0.8836 (3)	0.66881 (14)	0.81965 (15)	0.0344 (7)	
C8	0.9882 (3)	0.70185 (15)	0.82297 (16)	0.0372 (8)	
C9	0.9894 (3)	0.74058 (15)	0.78113 (17)	0.0392 (8)	
H9	1.057717	0.762959	0.781641	0.047*	
C10	0.8936 (3)	0.74789 (15)	0.73821 (16)	0.0371 (8)	
H10	0.898217	0.775162	0.710376	0.044*	
C11	0.7676 (4)	0.59493 (15)	0.85110 (17)	0.0420 (8)	
H11	0.760653	0.566427	0.877238	0.050*	
C12	0.8689 (4)	0.62497 (15)	0.85710 (17)	0.0410 (8)	
H12	0.931331	0.616638	0.886880	0.049*	
C13	0.5409 (4)	0.53348 (16)	0.84122 (18)	0.0430 (9)	
C14	0.6087 (4)	0.48711 (17)	0.84560 (19)	0.0476 (9)	
H14	0.666919	0.481392	0.819985	0.057*	
C15	0.5927 (4)	0.44903 (19)	0.8869 (2)	0.0558 (11)	
H15	0.638969	0.417219	0.889383	0.067*	
C16	0.5082 (5)	0.4578 (2)	0.9246 (2)	0.0623 (13)	
H16	0.497500	0.432103	0.953341	0.075*	
C17	0.4402 (5)	0.5034 (2)	0.9205 (2)	0.0612 (13)	
H17	0.383720	0.509189	0.946977	0.073*	
C18	0.4524 (4)	0.54113 (19)	0.8785 (2)	0.0530 (10)	
H18	0.402162	0.571714	0.874737	0.064*	
C19	1.0915 (3)	0.69433 (15)	0.86768 (17)	0.0398 (8)	
C20	1.2076 (4)	0.69364 (18)	0.8510 (2)	0.0498 (10)	
H20	1.218902	0.698542	0.811734	0.060*	
C21	1.3058 (4)	0.6857 (2)	0.8924 (2)	0.0597 (12)	
H21	1.384291	0.685222	0.881279	0.072*	
C22	1.2903 (5)	0.67864 (19)	0.9492 (2)	0.0616 (13)	
H22	1.357999	0.672767	0.977012	0.074*	
C23	1.1762 (5)	0.68000 (18)	0.9662 (2)	0.0552 (11)	
H23	1.166012	0.675357	1.005603	0.066*	
C24	1.0770 (4)	0.68816 (16)	0.92563 (18)	0.0448 (9)	
H24	0.999071	0.689531	0.937390	0.054*	
C25	0.7630 (4)	0.78075 (15)	0.59100 (17)	0.0393 (8)	
H25	0.776432	0.748309	0.571790	0.047*	
C26	0.8000 (4)	0.82847 (17)	0.56893 (17)	0.0417 (8)	
H26	0.836290	0.827849	0.534316	0.050*	
C27	0.7860 (3)	0.87685 (16)	0.59549 (16)	0.0376 (8)	
C28	0.7286 (3)	0.87611 (15)	0.64690 (15)	0.0355 (7)	
C29	0.6894 (3)	0.82666 (15)	0.66521 (15)	0.0336 (7)	
C30	0.6204 (3)	0.82295 (14)	0.71241 (15)	0.0336 (7)	
C31	0.5873 (3)	0.86886 (15)	0.74036 (15)	0.0353 (7)	
C32	0.5039 (3)	0.86268 (16)	0.78164 (16)	0.0385 (8)	
C33	0.4640 (4)	0.81197 (18)	0.79079 (17)	0.0448 (9)	
H33	0.406076	0.806748	0.816649	0.054*	
C34	0.5064 (4)	0.76813 (17)	0.76310 (17)	0.0417 (8)	
H34	0.479814	0.733505	0.772105	0.050*	
C35	0.7016 (4)	0.92207 (16)	0.67916 (16)	0.0411 (8)	
H35	0.732044	0.955821	0.669557	0.049*	
C36	0.6333 (4)	0.91869 (15)	0.72329 (16)	0.0404 (8)	
H36	0.615865	0.950239	0.743172	0.048*	
C37	0.8235 (3)	0.92742 (16)	0.57065 (17)	0.0406 (8)	
C38	0.7885 (4)	0.93826 (17)	0.51282 (18)	0.0452 (9)	
H38	0.746110	0.912285	0.488768	0.054*	
C39	0.8163 (5)	0.98768 (19)	0.4904 (2)	0.0546 (11)	
H39	0.791980	0.995228	0.450898	0.066*	
C40	0.8775 (5)	1.0252 (2)	0.5241 (2)	0.0642 (13)	
H40	0.893511	1.059098	0.508530	0.077*	
C41	0.9166 (5)	1.0136 (2)	0.5815 (2)	0.0694 (14)	
H41	0.961487	1.039373	0.604837	0.083*	
C42	0.8908 (4)	0.9650 (2)	0.6047 (2)	0.0553 (11)	
H42	0.918661	0.957125	0.643728	0.066*	
C43	0.4570 (4)	0.91008 (17)	0.80923 (17)	0.0438 (9)	
C44	0.5344 (4)	0.94603 (19)	0.84093 (18)	0.0497 (10)	
H44	0.617986	0.938852	0.847674	0.060*	
C45	0.4885 (5)	0.9920 (2)	0.8623 (2)	0.0669 (14)	
H45	0.540387	1.015941	0.884830	0.080*	
C46	0.3678 (6)	1.0033 (2)	0.8512 (3)	0.0749 (16)	
H46	0.337699	1.035581	0.865191	0.090*	
C47	0.2904 (5)	0.9684 (3)	0.8202 (3)	0.0775 (17)	
H47	0.207405	0.976544	0.812452	0.093*	
C48	0.3352 (4)	0.9214 (2)	0.8003 (2)	0.0599 (12)	
H48	0.281748	0.896444	0.780217	0.072*	
C49	0.8241 (3)	0.64466 (15)	0.63394 (16)	0.0369 (8)	
H49	0.883090	0.655760	0.664172	0.044*	
C50	0.8531 (3)	0.60596 (15)	0.59594 (16)	0.0370 (8)	
H50	0.931940	0.591600	0.600143	0.044*	
C51	0.7700 (3)	0.58771 (14)	0.55201 (15)	0.0328 (7)	
C52	0.6528 (3)	0.61036 (14)	0.54691 (15)	0.0316 (7)	
C53	0.6319 (3)	0.64999 (13)	0.58581 (15)	0.0308 (7)	
C54	0.5186 (3)	0.67625 (14)	0.58226 (15)	0.0327 (7)	
C55	0.4255 (3)	0.66234 (14)	0.53978 (15)	0.0329 (7)	
C56	0.3169 (3)	0.69295 (15)	0.53654 (17)	0.0373 (8)	
C57	0.3126 (4)	0.73284 (17)	0.57683 (19)	0.0442 (9)	
H57	0.242483	0.754196	0.575696	0.053*	
C58	0.4091 (3)	0.74269 (16)	0.61940 (18)	0.0424 (9)	
H58	0.401972	0.770082	0.646843	0.051*	
C59	0.5533 (3)	0.59306 (15)	0.50671 (16)	0.0357 (7)	
H59	0.563429	0.563724	0.482060	0.043*	
C60	0.4445 (3)	0.61795 (15)	0.50324 (16)	0.0354 (7)	
H60	0.380459	0.605677	0.476192	0.042*	
C61	0.8004 (3)	0.54451 (15)	0.51347 (16)	0.0358 (7)	
C62	0.8472 (3)	0.49669 (16)	0.53789 (18)	0.0410 (8)	
H62	0.861949	0.493119	0.578564	0.049*	
C63	0.8717 (4)	0.45475 (17)	0.5028 (2)	0.0485 (10)	
H63	0.902871	0.422302	0.519458	0.058*	
C64	0.8514 (4)	0.45979 (19)	0.4442 (2)	0.0509 (10)	
H64	0.864681	0.430264	0.420356	0.061*	
C65	0.8114 (4)	0.5080 (2)	0.41955 (19)	0.0542 (11)	
H65	0.802035	0.512000	0.378877	0.065*	
C66	0.7852 (4)	0.55008 (18)	0.45419 (17)	0.0453 (9)	
H66	0.756653	0.582820	0.437249	0.054*	
C67	0.2124 (3)	0.68412 (15)	0.49285 (18)	0.0393 (8)	
C68	0.2249 (4)	0.67567 (16)	0.43524 (18)	0.0425 (8)	
H68	0.302622	0.674836	0.423196	0.051*	
C69	0.1243 (4)	0.66842 (18)	0.3950 (2)	0.0515 (10)	
H69	0.133435	0.662434	0.355684	0.062*	
C70	0.0104 (4)	0.66996 (19)	0.4125 (2)	0.0551 (11)	
H70	−0.058344	0.664926	0.385150	0.066*	
C71	−0.0030 (4)	0.67879 (17)	0.4697 (2)	0.0536 (11)	
H71	−0.081008	0.680011	0.481433	0.064*	
C72	0.0967 (3)	0.68585 (16)	0.5098 (2)	0.0453 (9)	
H72	0.086903	0.691887	0.549084	0.054*	
Co1	0.65174 (5)	0.71899 (2)	0.67893 (2)	0.03151 (17)	
N1	0.6003 (3)	0.66029 (12)	0.72480 (13)	0.0348 (6)	
N2	0.7953 (3)	0.71751 (11)	0.73518 (13)	0.0333 (6)	
N3	0.7088 (3)	0.77952 (12)	0.63882 (13)	0.0343 (6)	
N4	0.5829 (3)	0.77309 (12)	0.72446 (13)	0.0350 (6)	
N5	0.7153 (3)	0.66689 (12)	0.62928 (13)	0.0317 (6)	
N6	0.5105 (3)	0.71465 (12)	0.62244 (14)	0.0356 (6)	
F1	0.7023 (3)	0.70768 (14)	0.47885 (13)	0.0702 (8)	
F2	0.5998 (3)	0.78510 (16)	0.47147 (16)	0.0821 (10)	
F3	0.7786 (3)	0.77811 (14)	0.43789 (14)	0.0705 (9)	
F4	0.6076 (3)	0.78566 (14)	0.37517 (14)	0.0766 (10)	
F5	0.7095 (3)	0.70887 (17)	0.38305 (15)	0.0810 (10)	
F6	0.5304 (3)	0.71560 (14)	0.41707 (13)	0.0715 (9)	
P1	0.65426 (9)	0.74674 (5)	0.42702 (5)	0.0482 (3)	
F7	0.1732 (3)	0.82570 (15)	0.72944 (19)	0.0954 (12)	
F8	0.1590 (2)	0.90313 (11)	0.67956 (13)	0.0646 (8)	
F9	−0.0012 (2)	0.84994 (12)	0.68175 (14)	0.0641 (7)	
F10	0.0829 (4)	0.85907 (17)	0.60049 (16)	0.0907 (11)	
F11	0.0985 (3)	0.78177 (13)	0.6499 (2)	0.0893 (12)	
F12	0.2617 (3)	0.83455 (14)	0.6485 (2)	0.1071 (14)	
P2	0.12975 (10)	0.84315 (5)	0.66478 (6)	0.0546 (3)	
F13	0.0178 (3)	0.52703 (16)	0.67741 (13)	0.0883 (10)	
F14	0.1488 (5)	0.5855 (2)	0.7846 (2)	0.1456 (16)	
F15A	−0.0113 (13)	0.5394 (7)	0.7751 (6)	0.118 (3)	0.303 (5)
F15B	0.0464 (8)	0.5084 (3)	0.7714 (3)	0.1140 (18)	0.697 (5)
F16A	0.1522 (16)	0.5012 (5)	0.7440 (7)	0.121 (2)	0.303 (5)
F16B	0.2034 (5)	0.5305 (3)	0.7229 (3)	0.1017 (16)	0.697 (5)
F17A	0.1890 (11)	0.5803 (7)	0.6993 (6)	0.118 (3)	0.303 (5)
F17B	0.1091 (8)	0.6030 (3)	0.6892 (3)	0.1148 (18)	0.697 (5)
F18A	0.0146 (12)	0.6121 (4)	0.7185 (5)	0.113 (2)	0.303 (5)
F18B	−0.0489 (6)	0.5801 (3)	0.7374 (3)	0.1184 (18)	0.697 (5)
P3	0.08027 (17)	0.55560 (6)	0.73172 (6)	0.0755 (5)	
O1	0.2529 (5)	0.6951 (3)	0.7055 (3)	0.1140 (19)	
H1A	0.235 (8)	0.666 (2)	0.722 (4)	0.171*	
H1B	0.186 (5)	0.705 (4)	0.685 (4)	0.171*	

### Atomic displacement parameters (Å^2^)

**Table:**

	*U*^11^	*U*^22^	*U*^33^	*U*^12^	*U*^13^	*U*^23^
C1	0.0364 (19)	0.043 (2)	0.047 (2)	−0.0070 (16)	0.0041 (16)	−0.0024 (16)
C2	0.038 (2)	0.045 (2)	0.051 (2)	−0.0085 (17)	0.0065 (16)	−0.0037 (18)
C3	0.047 (2)	0.0334 (18)	0.0419 (19)	−0.0061 (15)	0.0133 (16)	−0.0050 (15)
C4	0.0432 (19)	0.0307 (17)	0.0366 (17)	−0.0028 (15)	0.0080 (15)	−0.0044 (14)
C5	0.0342 (17)	0.0308 (16)	0.0370 (17)	0.0017 (13)	0.0077 (14)	−0.0049 (13)
C6	0.0337 (17)	0.0295 (16)	0.0360 (17)	0.0021 (13)	0.0057 (13)	−0.0055 (13)
C7	0.0355 (17)	0.0304 (16)	0.0374 (17)	0.0010 (14)	0.0051 (14)	−0.0056 (14)
C8	0.0363 (18)	0.0357 (18)	0.0390 (18)	0.0025 (15)	0.0019 (14)	−0.0074 (15)
C9	0.0357 (18)	0.0365 (19)	0.045 (2)	−0.0051 (15)	0.0047 (15)	−0.0021 (15)
C10	0.0364 (18)	0.0341 (18)	0.0404 (19)	−0.0044 (14)	0.0037 (15)	−0.0006 (14)
C11	0.052 (2)	0.0317 (18)	0.042 (2)	−0.0030 (16)	0.0038 (17)	0.0031 (15)
C12	0.047 (2)	0.0338 (18)	0.0404 (19)	−0.0001 (16)	−0.0003 (16)	0.0009 (15)
C13	0.045 (2)	0.0393 (19)	0.045 (2)	−0.0108 (16)	0.0089 (16)	−0.0042 (16)
C14	0.048 (2)	0.044 (2)	0.051 (2)	−0.0096 (18)	0.0052 (18)	0.0020 (18)
C15	0.056 (3)	0.048 (2)	0.062 (3)	−0.013 (2)	−0.002 (2)	0.008 (2)
C16	0.069 (3)	0.064 (3)	0.052 (3)	−0.029 (3)	0.000 (2)	0.015 (2)
C17	0.071 (3)	0.063 (3)	0.053 (3)	−0.026 (3)	0.021 (2)	−0.006 (2)
C18	0.059 (3)	0.047 (2)	0.056 (2)	−0.013 (2)	0.020 (2)	−0.0057 (19)
C19	0.0374 (19)	0.0333 (18)	0.047 (2)	−0.0022 (15)	−0.0012 (16)	−0.0043 (15)
C20	0.039 (2)	0.047 (2)	0.063 (3)	0.0004 (17)	0.0015 (18)	−0.006 (2)
C21	0.040 (2)	0.053 (3)	0.082 (3)	0.0034 (19)	−0.005 (2)	−0.014 (2)
C22	0.052 (3)	0.045 (2)	0.079 (3)	0.004 (2)	−0.025 (2)	−0.005 (2)
C23	0.064 (3)	0.044 (2)	0.053 (2)	−0.004 (2)	−0.013 (2)	0.0004 (19)
C24	0.045 (2)	0.0359 (19)	0.051 (2)	−0.0025 (16)	−0.0055 (17)	−0.0027 (16)
C25	0.041 (2)	0.041 (2)	0.0380 (19)	0.0063 (15)	0.0094 (15)	−0.0003 (15)
C26	0.043 (2)	0.046 (2)	0.0382 (19)	0.0017 (16)	0.0099 (15)	−0.0009 (16)
C27	0.0348 (18)	0.0404 (19)	0.0370 (18)	0.0005 (15)	0.0019 (14)	0.0011 (15)
C28	0.0334 (17)	0.0374 (18)	0.0350 (17)	0.0018 (14)	0.0009 (14)	0.0006 (14)
C29	0.0307 (16)	0.0369 (18)	0.0328 (16)	0.0041 (14)	0.0023 (13)	−0.0019 (14)
C30	0.0319 (17)	0.0356 (18)	0.0328 (16)	0.0021 (14)	0.0014 (13)	−0.0008 (14)
C31	0.0346 (17)	0.0388 (19)	0.0317 (16)	0.0038 (14)	0.0015 (13)	−0.0038 (14)
C32	0.0368 (18)	0.044 (2)	0.0345 (17)	0.0033 (15)	0.0017 (14)	−0.0065 (15)
C33	0.042 (2)	0.054 (2)	0.040 (2)	−0.0024 (18)	0.0118 (16)	−0.0048 (17)
C34	0.042 (2)	0.0412 (19)	0.043 (2)	−0.0034 (16)	0.0110 (16)	−0.0015 (16)
C35	0.048 (2)	0.0366 (19)	0.0388 (19)	0.0003 (16)	0.0033 (16)	−0.0009 (15)
C36	0.049 (2)	0.0347 (18)	0.0377 (18)	0.0037 (16)	0.0041 (16)	−0.0033 (15)
C37	0.0382 (19)	0.041 (2)	0.044 (2)	−0.0007 (15)	0.0093 (15)	−0.0009 (16)
C38	0.051 (2)	0.044 (2)	0.043 (2)	0.0030 (18)	0.0130 (17)	0.0009 (17)
C39	0.071 (3)	0.048 (2)	0.048 (2)	0.005 (2)	0.020 (2)	0.0078 (19)
C40	0.085 (4)	0.049 (3)	0.064 (3)	−0.008 (2)	0.027 (3)	0.000 (2)
C41	0.079 (4)	0.062 (3)	0.070 (3)	−0.029 (3)	0.019 (3)	−0.009 (3)
C42	0.057 (3)	0.056 (3)	0.053 (2)	−0.015 (2)	0.010 (2)	−0.002 (2)
C43	0.045 (2)	0.049 (2)	0.0397 (19)	0.0013 (17)	0.0107 (16)	−0.0102 (17)
C44	0.050 (2)	0.058 (3)	0.041 (2)	0.000 (2)	0.0060 (17)	−0.0137 (19)
C45	0.072 (3)	0.067 (3)	0.065 (3)	−0.012 (3)	0.022 (3)	−0.031 (3)
C46	0.076 (4)	0.066 (3)	0.087 (4)	0.005 (3)	0.028 (3)	−0.034 (3)
C47	0.057 (3)	0.082 (4)	0.096 (4)	0.016 (3)	0.022 (3)	−0.028 (3)
C48	0.046 (2)	0.067 (3)	0.068 (3)	0.001 (2)	0.014 (2)	−0.025 (2)
C49	0.0300 (17)	0.0394 (19)	0.0405 (18)	0.0048 (14)	0.0010 (14)	−0.0018 (15)
C50	0.0284 (16)	0.0383 (18)	0.0446 (19)	0.0084 (14)	0.0057 (14)	0.0016 (15)
C51	0.0300 (16)	0.0328 (17)	0.0361 (17)	0.0053 (13)	0.0067 (13)	0.0044 (14)
C52	0.0283 (16)	0.0328 (16)	0.0340 (16)	0.0057 (13)	0.0045 (13)	0.0040 (13)
C53	0.0281 (16)	0.0286 (16)	0.0357 (17)	0.0041 (13)	0.0036 (13)	0.0044 (13)
C54	0.0298 (16)	0.0297 (16)	0.0390 (17)	0.0045 (13)	0.0063 (13)	−0.0001 (13)
C55	0.0258 (15)	0.0329 (17)	0.0396 (18)	0.0059 (13)	0.0023 (13)	0.0037 (14)
C56	0.0287 (17)	0.0361 (18)	0.047 (2)	0.0056 (14)	0.0031 (14)	0.0035 (15)
C57	0.0332 (18)	0.044 (2)	0.054 (2)	0.0123 (16)	−0.0005 (16)	−0.0041 (18)
C58	0.0354 (19)	0.042 (2)	0.049 (2)	0.0125 (16)	0.0021 (16)	−0.0072 (17)
C59	0.0330 (17)	0.0353 (18)	0.0380 (18)	0.0062 (14)	0.0013 (14)	−0.0015 (14)
C60	0.0279 (16)	0.0373 (18)	0.0398 (18)	0.0039 (14)	−0.0007 (13)	−0.0002 (15)
C61	0.0280 (16)	0.0392 (19)	0.0404 (18)	0.0073 (14)	0.0050 (14)	−0.0025 (15)
C62	0.0371 (19)	0.0384 (19)	0.048 (2)	0.0066 (15)	0.0083 (16)	0.0017 (16)
C63	0.041 (2)	0.039 (2)	0.067 (3)	0.0078 (17)	0.0133 (19)	−0.0022 (19)
C64	0.0326 (19)	0.055 (2)	0.066 (3)	0.0083 (18)	0.0083 (18)	−0.018 (2)
C65	0.043 (2)	0.074 (3)	0.045 (2)	0.018 (2)	0.0034 (17)	−0.012 (2)
C66	0.040 (2)	0.054 (2)	0.042 (2)	0.0165 (18)	0.0045 (16)	−0.0006 (17)
C67	0.0291 (17)	0.0308 (17)	0.056 (2)	0.0043 (14)	−0.0011 (15)	0.0028 (16)
C68	0.0334 (18)	0.043 (2)	0.049 (2)	0.0037 (15)	−0.0016 (16)	0.0079 (17)
C69	0.049 (2)	0.048 (2)	0.054 (2)	0.0029 (19)	−0.0098 (19)	0.0081 (19)
C70	0.039 (2)	0.049 (2)	0.072 (3)	−0.0013 (18)	−0.016 (2)	0.013 (2)
C71	0.0287 (19)	0.040 (2)	0.089 (3)	0.0037 (16)	−0.002 (2)	0.006 (2)
C72	0.0306 (18)	0.041 (2)	0.064 (3)	0.0064 (15)	0.0042 (17)	−0.0010 (18)
Co1	0.0292 (3)	0.0306 (3)	0.0345 (3)	0.0026 (2)	0.0030 (2)	−0.0013 (2)
N1	0.0307 (14)	0.0323 (15)	0.0411 (16)	0.0006 (12)	0.0029 (12)	−0.0025 (12)
N2	0.0321 (15)	0.0323 (15)	0.0356 (15)	−0.0016 (11)	0.0049 (12)	−0.0019 (11)
N3	0.0327 (15)	0.0353 (16)	0.0349 (15)	0.0038 (12)	0.0034 (12)	−0.0009 (12)
N4	0.0345 (15)	0.0353 (15)	0.0349 (15)	0.0010 (12)	0.0035 (12)	−0.0010 (12)
N5	0.0263 (13)	0.0314 (14)	0.0370 (15)	0.0038 (11)	0.0024 (11)	0.0007 (12)
N6	0.0314 (15)	0.0331 (15)	0.0422 (16)	0.0067 (12)	0.0046 (12)	−0.0014 (12)
F1	0.0608 (17)	0.083 (2)	0.0634 (17)	−0.0028 (15)	−0.0056 (14)	0.0286 (15)
F2	0.0568 (18)	0.109 (3)	0.078 (2)	0.0160 (17)	−0.0011 (16)	−0.0121 (19)
F3	0.0454 (15)	0.094 (2)	0.0692 (18)	−0.0200 (14)	−0.0024 (13)	0.0168 (16)
F4	0.0611 (18)	0.095 (2)	0.0685 (19)	−0.0068 (16)	−0.0116 (15)	0.0352 (17)
F5	0.0593 (18)	0.115 (3)	0.0678 (19)	0.0129 (18)	0.0040 (15)	−0.0164 (18)
F6	0.0462 (15)	0.102 (2)	0.0642 (18)	−0.0228 (15)	0.0000 (13)	0.0158 (16)
P1	0.0321 (5)	0.0658 (7)	0.0457 (6)	−0.0021 (5)	0.0007 (4)	0.0134 (5)
F7	0.073 (2)	0.074 (2)	0.130 (3)	−0.0179 (17)	−0.029 (2)	0.026 (2)
F8	0.0610 (17)	0.0442 (15)	0.090 (2)	−0.0024 (12)	0.0151 (15)	−0.0051 (13)
F9	0.0478 (14)	0.0667 (17)	0.0801 (19)	0.0022 (13)	0.0170 (13)	0.0018 (14)
F10	0.097 (3)	0.106 (3)	0.073 (2)	−0.022 (2)	0.0282 (19)	−0.0089 (19)
F11	0.079 (2)	0.0545 (18)	0.140 (3)	−0.0101 (15)	0.037 (2)	−0.0263 (19)
F12	0.0606 (19)	0.0581 (18)	0.212 (4)	0.0066 (15)	0.055 (2)	−0.008 (2)
P2	0.0403 (6)	0.0423 (6)	0.0830 (8)	0.0009 (4)	0.0152 (5)	−0.0074 (5)
F13	0.095 (2)	0.111 (2)	0.0591 (17)	0.024 (2)	0.0099 (16)	0.0028 (17)
F14	0.194 (4)	0.137 (3)	0.098 (3)	−0.002 (3)	−0.015 (3)	−0.031 (3)
F15A	0.143 (5)	0.126 (5)	0.085 (4)	0.022 (5)	0.019 (4)	0.003 (4)
F15B	0.144 (4)	0.125 (4)	0.073 (3)	0.020 (3)	0.013 (3)	0.028 (3)
F16A	0.142 (5)	0.122 (5)	0.095 (4)	0.025 (4)	0.001 (4)	0.002 (4)
F16B	0.092 (3)	0.113 (4)	0.096 (3)	0.012 (3)	−0.004 (3)	−0.015 (3)
F17A	0.133 (5)	0.120 (5)	0.093 (4)	−0.012 (5)	−0.020 (4)	0.008 (4)
F17B	0.146 (4)	0.091 (3)	0.110 (3)	−0.013 (3)	0.025 (3)	−0.006 (3)
F18A	0.138 (5)	0.104 (4)	0.098 (4)	0.026 (4)	0.017 (4)	0.004 (4)
F18B	0.147 (4)	0.127 (4)	0.085 (3)	0.061 (3)	0.031 (3)	−0.005 (3)
P3	0.1056 (12)	0.0701 (9)	0.0491 (7)	0.0314 (8)	0.0027 (7)	−0.0029 (6)
O1	0.097 (4)	0.114 (4)	0.140 (5)	0.022 (3)	0.053 (4)	0.037 (4)

### Geometric parameters (Å, º)

**Table:**

C1—H1	0.9500	C44—C45	1.381 (7)
C1—C2	1.395 (6)	C45—H45	0.9500
C1—N1	1.339 (5)	C45—C46	1.379 (8)
C2—H2	0.9500	C46—H46	0.9500
C2—C3	1.371 (6)	C46—C47	1.377 (9)
C3—C4	1.421 (5)	C47—H47	0.9500
C3—C13	1.483 (5)	C47—C48	1.385 (7)
C4—C5	1.398 (5)	C48—H48	0.9500
C4—C11	1.423 (6)	C49—H49	0.9500
C5—C6	1.424 (5)	C49—C50	1.381 (5)
C5—N1	1.363 (5)	C49—N5	1.336 (5)
C6—C7	1.396 (5)	C50—H50	0.9500
C6—N2	1.357 (5)	C50—C51	1.380 (5)
C7—C8	1.432 (5)	C51—C52	1.426 (5)
C7—C12	1.428 (5)	C51—C61	1.476 (5)
C8—C9	1.380 (6)	C52—C53	1.386 (5)
C8—C19	1.477 (5)	C52—C59	1.437 (5)
C9—H9	0.9500	C53—C54	1.427 (5)
C9—C10	1.391 (5)	C53—N5	1.363 (4)
C10—H10	0.9500	C54—C55	1.396 (5)
C10—N2	1.336 (5)	C54—N6	1.356 (5)
C11—H11	0.9500	C55—C56	1.435 (5)
C11—C12	1.357 (6)	C55—C60	1.435 (5)
C12—H12	0.9500	C56—C57	1.379 (6)
C13—C14	1.387 (6)	C56—C67	1.476 (5)
C13—C18	1.413 (6)	C57—H57	0.9500
C14—H14	0.9500	C57—C58	1.402 (6)
C14—C15	1.385 (6)	C58—H58	0.9500
C15—H15	0.9500	C58—N6	1.334 (5)
C15—C16	1.390 (8)	C59—H59	0.9500
C16—H16	0.9500	C59—C60	1.365 (5)
C16—C17	1.372 (8)	C60—H60	0.9500
C17—H17	0.9500	C61—C62	1.402 (5)
C17—C18	1.383 (7)	C61—C66	1.384 (5)
C18—H18	0.9500	C62—H62	0.9500
C19—C20	1.406 (6)	C62—C63	1.382 (6)
C19—C24	1.394 (6)	C63—H63	0.9500
C20—H20	0.9500	C63—C64	1.367 (7)
C20—C21	1.391 (7)	C64—H64	0.9500
C21—H21	0.9500	C64—C65	1.390 (7)
C21—C22	1.372 (8)	C65—H65	0.9500
C22—H22	0.9500	C65—C66	1.384 (6)
C22—C23	1.388 (8)	C66—H66	0.9500
C23—H23	0.9500	C67—C68	1.388 (6)
C23—C24	1.388 (6)	C67—C72	1.406 (5)
C24—H24	0.9500	C68—H68	0.9500
C25—H25	0.9500	C68—C69	1.392 (6)
C25—C26	1.387 (6)	C69—H69	0.9500
C25—N3	1.338 (5)	C69—C70	1.391 (7)
C26—H26	0.9500	C70—H70	0.9500
C26—C27	1.381 (6)	C70—C71	1.381 (8)
C27—C28	1.432 (5)	C71—H71	0.9500
C27—C37	1.478 (5)	C71—C72	1.383 (6)
C28—C29	1.400 (5)	C72—H72	0.9500
C28—C35	1.430 (5)	Co1—N1	1.950 (3)
C29—C30	1.428 (5)	Co1—N2	1.954 (3)
C29—N3	1.363 (5)	Co1—N3	1.934 (3)
C30—C31	1.396 (5)	Co1—N4	1.942 (3)
C30—N4	1.359 (5)	Co1—N5	1.941 (3)
C31—C32	1.434 (5)	Co1—N6	1.940 (3)
C31—C36	1.427 (5)	F1—P1	1.600 (3)
C32—C33	1.373 (6)	F2—P1	1.592 (4)
C32—C43	1.479 (5)	F3—P1	1.596 (3)
C33—H33	0.9500	F4—P1	1.595 (3)
C33—C34	1.389 (6)	F5—P1	1.581 (4)
C34—H34	0.9500	F6—P1	1.588 (3)
C34—N4	1.327 (5)	F7—P2	1.593 (4)
C35—H35	0.9500	F8—P2	1.569 (3)
C35—C36	1.362 (6)	F9—P2	1.579 (3)
C36—H36	0.9500	F10—P2	1.583 (4)
C37—C38	1.389 (6)	F11—P2	1.607 (3)
C37—C42	1.394 (6)	F12—P2	1.590 (3)
C38—H38	0.9500	F13—P3	1.550 (4)
C38—C39	1.396 (6)	F14—P3	1.567 (4)
C39—H39	0.9500	F15A—P3	1.583 (9)
C39—C40	1.359 (7)	F15B—P3	1.580 (6)
C40—H40	0.9500	F16A—P3	1.593 (9)
C40—C41	1.392 (8)	F16B—P3	1.557 (6)
C41—H41	0.9500	F17A—P3	1.636 (9)
C41—C42	1.380 (7)	F17B—P3	1.608 (6)
C42—H42	0.9500	F18A—P3	1.610 (7)
C43—C44	1.400 (6)	F18B—P3	1.596 (5)
C43—C48	1.389 (6)	O1—H1A	0.872 (5)
C44—H44	0.9500	O1—H1B	0.870 (5)
			
Cg1···Cg2^i^	3.707 (3)		
			
C2—C1—H1	119.2	C53—C52—C59	117.8 (3)
N1—C1—H1	119.2	C52—C53—C54	120.9 (3)
N1—C1—C2	121.6 (4)	N5—C53—C52	123.8 (3)
C1—C2—H2	119.4	N5—C53—C54	115.3 (3)
C3—C2—C1	121.3 (4)	C55—C54—C53	120.8 (3)
C3—C2—H2	119.4	N6—C54—C53	114.9 (3)
C2—C3—C4	117.8 (4)	N6—C54—C55	124.3 (3)
C2—C3—C13	122.7 (4)	C54—C55—C56	117.5 (3)
C4—C3—C13	119.4 (4)	C54—C55—C60	117.7 (3)
C3—C4—C11	124.7 (4)	C60—C55—C56	124.8 (3)
C5—C4—C3	118.0 (4)	C55—C56—C67	123.7 (3)
C5—C4—C11	117.3 (3)	C57—C56—C55	116.9 (3)
C4—C5—C6	120.9 (3)	C57—C56—C67	119.4 (3)
N1—C5—C4	122.9 (3)	C56—C57—H57	119.2
N1—C5—C6	116.2 (3)	C56—C57—C58	121.7 (3)
C7—C6—C5	120.8 (3)	C58—C57—H57	119.2
N2—C6—C5	115.3 (3)	C57—C58—H58	119.1
N2—C6—C7	123.9 (3)	N6—C58—C57	121.8 (4)
C6—C7—C8	117.9 (3)	N6—C58—H58	119.1
C6—C7—C12	117.5 (3)	C52—C59—H59	119.3
C12—C7—C8	124.6 (3)	C60—C59—C52	121.3 (3)
C7—C8—C19	122.3 (3)	C60—C59—H59	119.3
C9—C8—C7	116.6 (3)	C55—C60—H60	119.5
C9—C8—C19	121.2 (4)	C59—C60—C55	121.1 (3)
C8—C9—H9	119.0	C59—C60—H60	119.5
C8—C9—C10	122.1 (4)	C62—C61—C51	118.8 (3)
C10—C9—H9	119.0	C66—C61—C51	121.9 (3)
C9—C10—H10	119.2	C66—C61—C62	119.3 (4)
N2—C10—C9	121.6 (3)	C61—C62—H62	120.0
N2—C10—H10	119.2	C63—C62—C61	120.0 (4)
C4—C11—H11	119.0	C63—C62—H62	120.0
C12—C11—C4	122.0 (4)	C62—C63—H63	119.9
C12—C11—H11	119.0	C64—C63—C62	120.2 (4)
C7—C12—H12	119.3	C64—C63—H63	119.9
C11—C12—C7	121.5 (4)	C63—C64—H64	119.9
C11—C12—H12	119.3	C63—C64—C65	120.2 (4)
C14—C13—C3	122.0 (4)	C65—C64—H64	119.9
C14—C13—C18	119.3 (4)	C64—C65—H65	120.0
C18—C13—C3	118.6 (4)	C66—C65—C64	120.0 (4)
C13—C14—H14	119.6	C66—C65—H65	120.0
C15—C14—C13	120.8 (4)	C61—C66—C65	120.0 (4)
C15—C14—H14	119.6	C61—C66—H66	120.0
C14—C15—H15	120.3	C65—C66—H66	120.0
C14—C15—C16	119.4 (5)	C68—C67—C56	122.0 (3)
C16—C15—H15	120.3	C68—C67—C72	119.0 (4)
C15—C16—H16	119.8	C72—C67—C56	119.0 (4)
C17—C16—C15	120.4 (4)	C67—C68—H68	119.8
C17—C16—H16	119.8	C67—C68—C69	120.5 (4)
C16—C17—H17	119.5	C69—C68—H68	119.8
C16—C17—C18	121.1 (5)	C68—C69—H69	120.1
C18—C17—H17	119.5	C70—C69—C68	119.8 (5)
C13—C18—H18	120.5	C70—C69—H69	120.1
C17—C18—C13	119.0 (5)	C69—C70—H70	119.9
C17—C18—H18	120.5	C71—C70—C69	120.2 (4)
C20—C19—C8	118.7 (4)	C71—C70—H70	119.9
C24—C19—C8	121.9 (4)	C70—C71—H71	119.9
C24—C19—C20	119.4 (4)	C70—C71—C72	120.2 (4)
C19—C20—H20	120.3	C72—C71—H71	119.9
C21—C20—C19	119.4 (5)	C67—C72—H72	119.8
C21—C20—H20	120.3	C71—C72—C67	120.3 (4)
C20—C21—H21	119.7	C71—C72—H72	119.8
C22—C21—C20	120.7 (5)	N1—Co1—N2	83.72 (13)
C22—C21—H21	119.7	N3—Co1—N1	175.62 (13)
C21—C22—H22	119.9	N3—Co1—N2	92.66 (13)
C21—C22—C23	120.3 (4)	N3—Co1—N4	84.01 (13)
C23—C22—H22	119.9	N3—Co1—N5	94.01 (13)
C22—C23—H23	120.0	N3—Co1—N6	90.31 (13)
C22—C23—C24	120.0 (5)	N4—Co1—N1	93.48 (13)
C24—C23—H23	120.0	N4—Co1—N2	89.70 (13)
C19—C24—H24	119.9	N5—Co1—N1	88.67 (13)
C23—C24—C19	120.1 (4)	N5—Co1—N2	93.26 (12)
C23—C24—H24	119.9	N5—Co1—N4	176.52 (13)
C26—C25—H25	119.4	N6—Co1—N1	93.44 (13)
N3—C25—H25	119.4	N6—Co1—N2	175.65 (13)
N3—C25—C26	121.3 (3)	N6—Co1—N4	93.77 (13)
C25—C26—H26	118.8	N6—Co1—N5	83.36 (12)
C27—C26—C25	122.3 (4)	C1—N1—C5	118.3 (3)
C27—C26—H26	118.8	C1—N1—Co1	129.6 (3)
C26—C27—C28	117.0 (3)	C5—N1—Co1	112.1 (2)
C26—C27—C37	121.5 (3)	C6—N2—Co1	112.7 (2)
C28—C27—C37	121.4 (3)	C10—N2—C6	118.0 (3)
C29—C28—C27	117.3 (3)	C10—N2—Co1	129.4 (3)
C29—C28—C35	117.2 (3)	C25—N3—C29	118.2 (3)
C35—C28—C27	125.4 (3)	C25—N3—Co1	129.4 (3)
C28—C29—C30	121.0 (3)	C29—N3—Co1	112.3 (2)
N3—C29—C28	123.8 (3)	C30—N4—Co1	112.0 (2)
N3—C29—C30	115.2 (3)	C34—N4—C30	118.0 (3)
C31—C30—C29	120.6 (3)	C34—N4—Co1	130.0 (3)
N4—C30—C29	115.5 (3)	C49—N5—C53	118.0 (3)
N4—C30—C31	123.7 (3)	C49—N5—Co1	129.2 (3)
C30—C31—C32	117.3 (3)	C53—N5—Co1	112.7 (2)
C30—C31—C36	117.7 (3)	C54—N6—Co1	113.2 (2)
C36—C31—C32	124.9 (3)	C58—N6—C54	117.8 (3)
C31—C32—C43	120.2 (4)	C58—N6—Co1	129.0 (3)
C33—C32—C31	117.2 (3)	F2—P1—F1	89.8 (2)
C33—C32—C43	122.4 (4)	F2—P1—F3	89.8 (2)
C32—C33—H33	119.2	F2—P1—F4	90.6 (2)
C32—C33—C34	121.5 (4)	F3—P1—F1	88.44 (17)
C34—C33—H33	119.2	F4—P1—F1	179.46 (19)
C33—C34—H34	119.0	F4—P1—F3	91.15 (17)
N4—C34—C33	122.1 (4)	F5—P1—F1	90.1 (2)
N4—C34—H34	119.0	F5—P1—F2	179.5 (2)
C28—C35—H35	119.1	F5—P1—F3	89.7 (2)
C36—C35—C28	121.7 (4)	F5—P1—F4	89.5 (2)
C36—C35—H35	119.1	F5—P1—F6	90.9 (2)
C31—C36—H36	119.2	F6—P1—F1	91.10 (17)
C35—C36—C31	121.5 (4)	F6—P1—F2	89.6 (2)
C35—C36—H36	119.2	F6—P1—F3	179.26 (18)
C38—C37—C27	119.4 (4)	F6—P1—F4	89.32 (17)
C38—C37—C42	119.6 (4)	F7—P2—F11	88.6 (2)
C42—C37—C27	121.1 (4)	F8—P2—F7	91.27 (19)
C37—C38—H38	120.3	F8—P2—F9	91.02 (16)
C37—C38—C39	119.3 (4)	F8—P2—F10	90.2 (2)
C39—C38—H38	120.3	F8—P2—F11	179.4 (2)
C38—C39—H39	119.4	F8—P2—F12	90.37 (18)
C40—C39—C38	121.1 (4)	F9—P2—F7	88.7 (2)
C40—C39—H39	119.4	F9—P2—F10	90.0 (2)
C39—C40—H40	120.2	F9—P2—F11	88.40 (18)
C39—C40—C41	119.6 (5)	F9—P2—F12	178.3 (2)
C41—C40—H40	120.2	F10—P2—F7	178.0 (2)
C40—C41—H41	119.8	F10—P2—F11	89.8 (2)
C42—C41—C40	120.5 (5)	F10—P2—F12	91.0 (3)
C42—C41—H41	119.8	F12—P2—F7	90.2 (3)
C37—C42—H42	120.1	F12—P2—F11	90.2 (2)
C41—C42—C37	119.8 (5)	F13—P3—F14	176.9 (3)
C41—C42—H42	120.1	F13—P3—F15A	98.2 (7)
C44—C43—C32	121.1 (4)	F13—P3—F15B	91.1 (3)
C48—C43—C32	119.6 (4)	F13—P3—F16A	85.5 (7)
C48—C43—C44	119.1 (4)	F13—P3—F16B	91.4 (3)
C43—C44—H44	120.2	F13—P3—F17A	94.8 (6)
C45—C44—C43	119.6 (4)	F13—P3—F17B	86.5 (3)
C45—C44—H44	120.2	F13—P3—F18A	95.6 (4)
C44—C45—H45	119.8	F13—P3—F18B	85.4 (3)
C46—C45—C44	120.4 (5)	F14—P3—F15A	84.9 (7)
C46—C45—H45	119.8	F14—P3—F15B	91.4 (4)
C45—C46—H46	119.6	F14—P3—F16A	94.4 (7)
C47—C46—C45	120.8 (5)	F14—P3—F17A	82.0 (6)
C47—C46—H46	119.6	F14—P3—F17B	91.1 (4)
C46—C47—H47	120.4	F14—P3—F18A	84.3 (4)
C46—C47—C48	119.1 (5)	F14—P3—F18B	96.6 (3)
C48—C47—H47	120.4	F15A—P3—F16A	91.2 (6)
C43—C48—H48	119.5	F15A—P3—F17A	166.9 (9)
C47—C48—C43	121.0 (5)	F15A—P3—F18A	91.7 (8)
C47—C48—H48	119.5	F15B—P3—F17B	177.3 (4)
C50—C49—H49	119.2	F15B—P3—F18B	87.3 (4)
N5—C49—H49	119.1	F16A—P3—F17A	91.0 (9)
N5—C49—C50	121.7 (3)	F16A—P3—F18A	176.7 (9)
C49—C50—H50	119.3	F16B—P3—F14	86.5 (3)
C51—C50—C49	121.4 (3)	F16B—P3—F15B	93.3 (4)
C51—C50—H50	119.3	F16B—P3—F17B	88.1 (4)
C50—C51—C52	117.7 (3)	F16B—P3—F18B	176.8 (3)
C50—C51—C61	120.8 (3)	F18A—P3—F17A	85.9 (5)
C52—C51—C61	121.4 (3)	F18B—P3—F17B	91.2 (4)
C51—C52—C59	124.8 (3)	H1A—O1—H1B	104.3 (13)
C53—C52—C51	117.3 (3)		
			
C1—C2—C3—C4	1.9 (6)	C33—C32—C43—C44	126.5 (5)
C1—C2—C3—C13	179.0 (4)	C33—C32—C43—C48	−58.2 (6)
C2—C1—N1—C5	−0.2 (6)	C33—C34—N4—C30	0.3 (6)
C2—C1—N1—Co1	−179.7 (3)	C33—C34—N4—Co1	−178.3 (3)
C2—C3—C4—C5	0.2 (5)	C35—C28—C29—C30	3.0 (5)
C2—C3—C4—C11	177.6 (4)	C35—C28—C29—N3	−179.9 (3)
C2—C3—C13—C14	118.2 (5)	C36—C31—C32—C33	176.6 (4)
C2—C3—C13—C18	−64.2 (5)	C36—C31—C32—C43	1.2 (6)
C3—C4—C5—C6	176.8 (3)	C37—C27—C28—C29	176.1 (3)
C3—C4—C5—N1	−2.4 (5)	C37—C27—C28—C35	0.1 (6)
C3—C4—C11—C12	−176.9 (4)	C37—C38—C39—C40	0.4 (7)
C3—C13—C14—C15	176.1 (4)	C38—C37—C42—C41	3.3 (7)
C3—C13—C18—C17	−174.3 (4)	C38—C39—C40—C41	2.1 (8)
C4—C3—C13—C14	−64.8 (5)	C39—C40—C41—C42	−1.8 (9)
C4—C3—C13—C18	112.8 (5)	C40—C41—C42—C37	−0.9 (8)
C4—C5—C6—C7	−0.3 (5)	C42—C37—C38—C39	−3.1 (6)
C4—C5—C6—N2	178.9 (3)	C43—C32—C33—C34	178.0 (4)
C4—C5—N1—C1	2.4 (5)	C43—C44—C45—C46	−1.9 (8)
C4—C5—N1—Co1	−177.9 (3)	C44—C43—C48—C47	2.7 (8)
C4—C11—C12—C7	1.0 (6)	C44—C45—C46—C47	1.9 (10)
C5—C4—C11—C12	0.5 (6)	C45—C46—C47—C48	0.4 (10)
C5—C6—C7—C8	179.1 (3)	C46—C47—C48—C43	−2.7 (10)
C5—C6—C7—C12	1.8 (5)	C48—C43—C44—C45	−0.4 (7)
C5—C6—N2—C10	−178.6 (3)	C49—C50—C51—C52	0.3 (5)
C5—C6—N2—Co1	0.0 (4)	C49—C50—C51—C61	−177.0 (3)
C6—C5—N1—C1	−176.8 (3)	C50—C49—N5—C53	0.5 (5)
C6—C5—N1—Co1	2.9 (4)	C50—C49—N5—Co1	176.9 (3)
C6—C7—C8—C9	−0.7 (5)	C50—C51—C52—C53	1.4 (5)
C6—C7—C8—C19	−179.0 (3)	C50—C51—C52—C59	−174.8 (3)
C6—C7—C12—C11	−2.1 (6)	C50—C51—C61—C62	53.0 (5)
C7—C6—N2—C10	0.6 (5)	C50—C51—C61—C66	−126.4 (4)
C7—C6—N2—Co1	179.2 (3)	C51—C52—C53—C54	177.5 (3)
C7—C8—C9—C10	0.8 (6)	C51—C52—C53—N5	−2.3 (5)
C7—C8—C19—C20	132.8 (4)	C51—C52—C59—C60	−177.9 (4)
C7—C8—C19—C24	−47.5 (5)	C51—C61—C62—C63	177.3 (4)
C8—C7—C12—C11	−179.3 (4)	C51—C61—C66—C65	−178.0 (4)
C8—C9—C10—N2	−0.3 (6)	C52—C51—C61—C62	−124.1 (4)
C8—C19—C20—C21	−178.9 (4)	C52—C51—C61—C66	56.4 (5)
C8—C19—C24—C23	178.6 (4)	C52—C53—C54—C55	0.5 (5)
C9—C8—C19—C20	−45.5 (5)	C52—C53—C54—N6	−179.2 (3)
C9—C8—C19—C24	134.3 (4)	C52—C53—N5—C49	1.4 (5)
C9—C10—N2—C6	−0.5 (5)	C52—C53—N5—Co1	−175.6 (3)
C9—C10—N2—Co1	−178.8 (3)	C52—C59—C60—C55	−0.1 (6)
C11—C4—C5—C6	−0.9 (5)	C53—C52—C59—C60	5.8 (5)
C11—C4—C5—N1	180.0 (3)	C53—C54—C55—C56	−175.9 (3)
C12—C7—C8—C9	176.5 (4)	C53—C54—C55—C60	5.2 (5)
C12—C7—C8—C19	−1.9 (6)	C53—C54—N6—C58	176.3 (3)
C13—C3—C4—C5	−177.0 (3)	C53—C54—N6—Co1	−5.5 (4)
C13—C3—C4—C11	0.5 (6)	C54—C53—N5—C49	−178.4 (3)
C13—C14—C15—C16	−0.6 (7)	C54—C53—N5—Co1	4.6 (4)
C14—C13—C18—C17	3.4 (7)	C54—C55—C56—C57	−1.6 (5)
C14—C15—C16—C17	0.9 (7)	C54—C55—C56—C67	177.9 (3)
C15—C16—C17—C18	1.1 (8)	C54—C55—C60—C59	−5.4 (5)
C16—C17—C18—C13	−3.2 (7)	C55—C54—N6—C58	−3.4 (6)
C18—C13—C14—C15	−1.6 (6)	C55—C54—N6—Co1	174.7 (3)
C19—C8—C9—C10	179.2 (4)	C55—C56—C57—C58	−0.8 (6)
C19—C20—C21—C22	0.0 (7)	C55—C56—C67—C68	−43.7 (6)
C20—C19—C24—C23	−1.7 (6)	C55—C56—C67—C72	138.2 (4)
C20—C21—C22—C23	−0.9 (7)	C56—C55—C60—C59	175.8 (4)
C21—C22—C23—C24	0.5 (7)	C56—C57—C58—N6	1.2 (7)
C22—C23—C24—C19	0.8 (6)	C56—C67—C68—C69	−178.9 (4)
C24—C19—C20—C21	1.3 (6)	C56—C67—C72—C71	178.7 (4)
C25—C26—C27—C28	−1.3 (6)	C57—C56—C67—C68	135.8 (4)
C25—C26—C27—C37	−178.6 (4)	C57—C56—C67—C72	−42.3 (5)
C26—C25—N3—C29	0.7 (6)	C57—C58—N6—C54	0.8 (6)
C26—C25—N3—Co1	−178.7 (3)	C57—C58—N6—Co1	−177.0 (3)
C26—C27—C28—C29	−1.3 (5)	C59—C52—C53—C54	−6.0 (5)
C26—C27—C28—C35	−177.3 (4)	C59—C52—C53—N5	174.2 (3)
C26—C27—C37—C38	48.8 (5)	C60—C55—C56—C57	177.2 (4)
C26—C27—C37—C42	−132.9 (4)	C60—C55—C56—C67	−3.2 (6)
C27—C28—C29—C30	−173.4 (3)	C61—C51—C52—C53	178.7 (3)
C27—C28—C29—N3	3.8 (5)	C61—C51—C52—C59	2.4 (5)
C27—C28—C35—C36	171.4 (4)	C61—C62—C63—C64	0.4 (6)
C27—C37—C38—C39	175.2 (4)	C62—C61—C66—C65	2.6 (6)
C27—C37—C42—C41	−174.9 (5)	C62—C63—C64—C65	3.1 (7)
C28—C27—C37—C38	−128.4 (4)	C63—C64—C65—C66	−3.8 (7)
C28—C27—C37—C42	49.9 (6)	C64—C65—C66—C61	1.0 (7)
C28—C29—C30—C31	1.9 (5)	C66—C61—C62—C63	−3.3 (6)
C28—C29—C30—N4	177.1 (3)	C67—C56—C57—C58	179.7 (4)
C28—C29—N3—C25	−3.5 (5)	C67—C68—C69—C70	0.4 (6)
C28—C29—N3—Co1	176.0 (3)	C68—C67—C72—C71	0.5 (6)
C28—C35—C36—C31	1.3 (6)	C68—C69—C70—C71	0.2 (7)
C29—C28—C35—C36	−4.6 (6)	C69—C70—C71—C72	−0.4 (7)
C29—C30—C31—C32	171.2 (3)	C70—C71—C72—C67	0.0 (6)
C29—C30—C31—C36	−5.1 (5)	C72—C67—C68—C69	−0.7 (6)
C29—C30—N4—C34	−171.9 (3)	N1—C1—C2—C3	−2.0 (6)
C29—C30—N4—Co1	7.0 (4)	N1—C5—C6—C7	178.9 (3)
C30—C29—N3—C25	173.8 (3)	N1—C5—C6—N2	−1.9 (4)
C30—C29—N3—Co1	−6.6 (4)	N2—C6—C7—C8	0.0 (5)
C30—C31—C32—C33	0.6 (5)	N2—C6—C7—C12	−177.4 (3)
C30—C31—C32—C43	−174.8 (3)	N3—C25—C26—C27	1.7 (6)
C30—C31—C36—C35	3.6 (6)	N3—C29—C30—C31	−175.5 (3)
C31—C30—N4—C34	3.2 (5)	N3—C29—C30—N4	−0.3 (5)
C31—C30—N4—Co1	−177.9 (3)	N4—C30—C31—C32	−3.7 (5)
C31—C32—C33—C34	2.7 (6)	N4—C30—C31—C36	−180.0 (3)
C31—C32—C43—C44	−58.4 (5)	N5—C49—C50—C51	−1.3 (6)
C31—C32—C43—C48	117.0 (5)	N5—C53—C54—C55	−179.7 (3)
C32—C31—C36—C35	−172.4 (4)	N5—C53—C54—N6	0.6 (5)
C32—C33—C34—N4	−3.3 (6)	N6—C54—C55—C56	3.8 (5)
C32—C43—C44—C45	175.0 (4)	N6—C54—C55—C60	−175.1 (3)
C32—C43—C48—C47	−172.8 (5)		

Symmetry code: (i) *x*+1, −*y*+3/2, *z*+1/2.

### Hydrogen-bond geometry (Å, º)

*Cg*1, *Cg*2, *Cg*3 and *Cg*4 are the centroids of atoms C19–C24, C67–C72, C37–C42 and N5/C49–C53, respectively.

**Table:**

*D*—H···*A*	*D*—H	H···*A*	*D*···*A*	*D*—H···*A*
C1—H1···O1	0.95	2.52	3.132 (7)	122
C2—H2···F17*A*	0.95	2.54	3.344 (14)	143
C9—H9···F7^ii^	0.95	2.46	3.300 (5)	148
C10—H10···F9^ii^	0.95	2.33	3.173 (5)	148
C10—H10···F11^ii^	0.95	2.80	3.383 (5)	120
C25—H25···F1	0.95	2.45	3.204 (5)	136
C26—H26···F3	0.95	2.59	3.297 (5)	132
C42—H42···F15*B*^iii^	0.95	2.36	3.096 (7)	134
C48—H48···F7	0.95	2.39	3.328 (6)	172
C48—H48···F8	0.95	2.59	3.278 (6)	130
C49—H49···F18*A*^ii^	0.95	2.13	2.850 (9)	132
C50—H50···F13^ii^	0.95	2.53	3.180 (5)	126
C50—H50···F17*B*^ii^	0.95	2.72	3.392 (10)	129
C57—H57···F11	0.95	2.61	3.349 (5)	135
C58—H58···F12	0.95	2.26	2.963 (5)	130
O1—H1*A*···F17*A*	0.87 (1)	2.25 (7)	2.965 (17)	139 (9)
O1—H1*A*···F17*B*	0.87 (1)	2.19 (8)	2.817 (10)	128 (8)
O1—H1*B*···F11	0.87 (1)	2.28 (7)	2.977 (7)	137 (8)
C17—H17···*Cg*3^iv^	0.95	2.80	3.525 (6)	134
C46—H46···*Cg*4^iii^	0.95	2.72	3.670 (6)	177
C63—H63···*Cg*5^v^	0.95	2.59	3.466 (5)	154

Symmetry codes: (ii) *x*+1, *y*, *z*; (iii) −*x*+1, *y*+1/2, −*z*+3/2; (iv) −*x*+1, *y*−1/2, −*z*+3/2; (v) −*x*+1, −*y*+1, −*z*+1.
